# Improving surgical outcomes

**Published:** 2008-12

**Authors:** Tony Walia, David Yorston

**Affiliations:** Ophthalmologist and Medical Director, PCEA Kikuyu Hospital, Kikuyu Eye Unit, Kikuyu, Kenya.; Consultant Ophthalmologist, Tennent Institute of Ophthalmology, Gartnavel Hospital, 1053 Great Western Road, Glasgow G12 0YN, UK.

**Figure:**
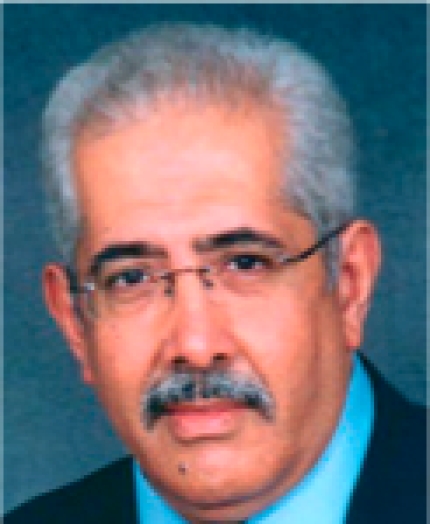


**Figure:**
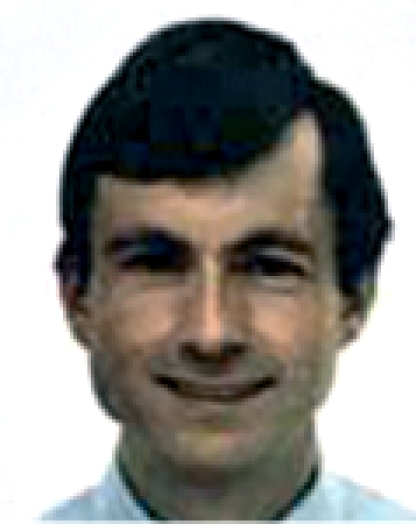


Outcomes of cataract surgery are worse than we would like them to be. Community-based studies show that up to 40% of eyes have a postoperative presenting vision of < 6/60.^1^ Eyes with intraocular lenses (IOLs) do better; however, it has been shown that even in prosperous middle-income countries, such as Venezuela, in 20% of pseudophakic eyes presenting vision was < 6/60 and in 15% best corrected vision was worse than 6/60.

Poor outcomes matter. Patients deserve improved vision whenever possible and poor outcomes deter prospective patients from coming for surgery and probably reduce their willingness to pay for their treatment – particularly if they have to pay in advance!

In this article, we offer some suggestions for improving the quality of cataract surgery. We admit that there is little evidence base for most of these suggestions and that some of them are controversial. However, we hope to stimulate debate.

## 1 Selection and training of ophthalmologists

Selection processes usually emphasise academic credentials rather than clinical or surgical skills. It is difficult to test surgical aptitude during a selection process; however, generic tests of hand-eye coordination do exist and are used routinely in the selection and training of pilots. Should we consider using similar tools to select ophthalmologists? At the very least, we should ensure that trainees have stereoscopic vision.

Selection is not always based on quality. Some postgraduate programmes do not even identify candidates who are interested in ophthalmology, because the country desperately needs ophthalmologists. The training of ophthalmic assistants in many countries in Africa offers another example. Originally, this training consisted of one year of clinical ophthalmology for everyone, after which suitable candidates were selected for another year of training in cataract surgery. However, to answer needs in personnel, training programmes now last eighteen months to two years and all students on the course are trained in cataract surgery, regardless of inclination or aptitude. The trainees' cataract surgical skills vary greatly and it is unlikely that this change has improved cataract outcomes.

Postgraduate training of eye surgeons should also have explicit targets for trainees, such as:

- number of operations that must be performed before trainees can qualify as ophthalmologists (e.g. in the UK, this number is 300, but most trainees perform more than 500 in practice)
- level of supervision: initially the trainee will be closely supervised by the trainer, but, by the conclusion of training, trainees should be able to operate on almost any cataract without supervision
- acceptable outcomes: e.g. simply performing the required number of operations would be insufficient if the trainee had a 25% vitreous loss rate.

## 2 Continuing medical education (CME)

In all medical disciplines, CME is vital. When ministries of health have so many claims on their small budgets, educating doctors is rarely a priority: after all, they have already received an expensive training. However, unless there is support for CME, the quality of care offered by specialists will deteriorate and this will reduce the value of the investment in their initial training.

CME is not just for doctors, but also for ophthalmic assistants and nurses. In the UK and the USA, qualified ophthalmologists must obtain a certain number of ‘CME points’ every year. Points can be obtained from private study. The process is administered by the Royal College of Ophthalmologists and the American Academy, respectively. This model, with its points system, may be one way in which ophthalmology institutions in affluent countries can assist low- and middle-income countries.

## 3 Innovation

At various stages in our careers, most of us have probably acquired a tip from another surgeon that enabled us to operate with greater confidence.

Eye surgery is not static and keeps improving. To improve our own surgery, we need to observe other surgeons and, occasionally, copy their techniques. This is easy in a large centre with multiple surgeons, but it is much more difficult if you are a surgeon working alone in a remote area. Those who work in larger centres should ensure that they can welcome other surgeons to observe and learn new techniques. This is also true for new materials and protocols, e.g. the use of cefuroxime in the prevention of endophthalmitis.^2^

## 4 Discipline

When we are under pressure to increase the numbers of cataract operations to 32 million per year by 2020, it is easy to focus on the quantity and lose sight of the quality.

Surgeons, and all eye workers, have to work in a systematic, disciplined way, so that all patients are fully assessed preoperatively and only those who are likely to benefit proceed to cataract surgery.

Because cataract surgery is performed so frequently, it can become routine, and we become careless. Doctors, nurses, and health managers need to sit together to develop robust processes and systems to ensure that every patient receives the best care and to minimise the risk of error. This may be as simple as ensuring that no patient is taken to theatre unless the eye for surgery is marked, or it may be as complex as a ten-page booklet that includes all preoperative and postoperative instructions.

## 5 Biometry

Many centres still use standard-power IOLs because they cannot perform biometry. Biometry equipment has become more portable and less expensive. Most surgeons should use it as a routine, even in outlying clinics. We are not aware of any randomised trials proving that preoperative biometry improves unaided postoperative vision. However, given that biometry is safe and inexpensive, it is difficult to justify withholding it from any patient. The prevalence of axial ametropia varies widely, and biometry is of greatest value in communities with the highest prevalence, e.g. in Asia. It will have a lesser impact where axial ametropia is less common, e.g. in sub-Saharan Africa.

**Figure:**
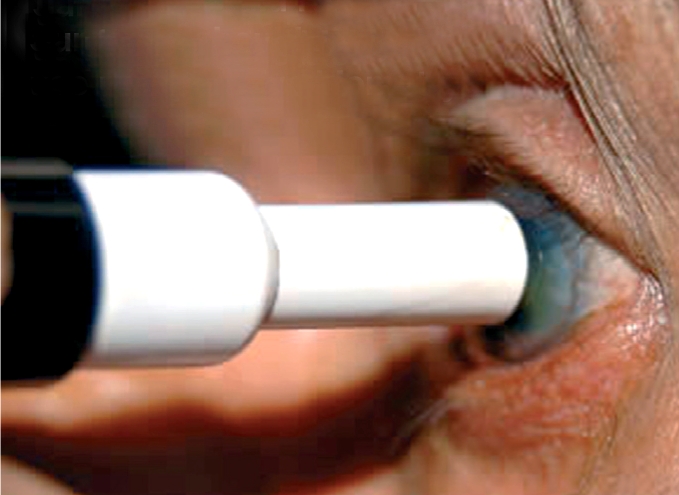
Surgeons should routinely use preoperative biometry

## 6 Equipment

It is difficult to obtain good results with inadequate equipment. If the operating microscope is broken, it is safer to cancel the operation than to proceed. This is frustrating for both surgeon and patient. However, the inconvenience of a cancelled operation is minor compared to the problems caused by complicated surgery. Ultimately, only surgeons can judge whether the equipment is adequate for their needs. What is acceptable to one may be unsuitable for another. For example, some eye instruments are designed for use by a right-handed surgeon. However, one of the authors is left-handed!

As ophthalmic surgery becomes more complex, regular maintenance of equipment is essential. Fortunately, this has been recognised by the VISION 2020 initiative, and training in equipment maintenance is available in some countries and regions.

**Figure:**
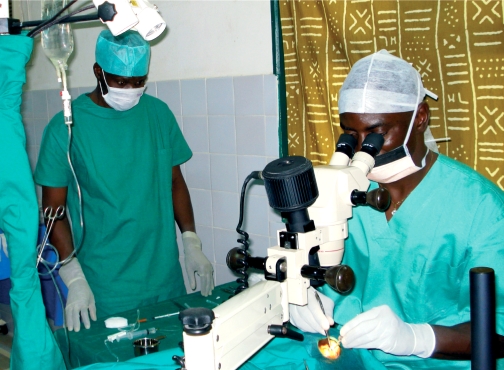
Cataract surgery: the more you operate, the better your surgical outcomes. IVORY COAST

## 7 Numbers

The more you do something, the better you do it – practice makes perfect. A surgeon who only operates on cataracts infrequently will have worse outcomes than a surgeon who operates every week. In most countries, almost all ophthalmologists do some cataract surgery. In countries where there are more than 50 ophthalmologists per million people, perhaps fewer of them should operate; this would allow operating ophthalmologists to increase their output and improve their outcomes. There is no agreed standard for the minimum number of operations an ophthalmologist should perform. However, we suggest that eye surgeons should operate at least once per week.

## 8 Audit

Prospective monitoring of outcomes was associated with an improvement in outcomes in three separate studies.^3^,^4^ Even a regular retrospective audit will identify problems and help us deal with them. If we do not set challenging outcome standards, we will remain in our ‘comfort zone’, but we are unlikely to improve our quality.^1^

## 9 Morbidity/mortality meetings

Although there are few cases of mortality in ophthalmology (we hope!), it is helpful to organise regular departmental meetings to discuss results and causes of poor outcomes. It is essential that these meetings are not vindictive or punitive. A single individual is rarely wholly responsible for a poor outcome. There is usually a sequence of errors, some of which are due to systemic failures in the institution. If these are to be corrected, the review must involve everyone, including surgeons, nurses, managers, and technical staff. The goal is not to find out what went wrong (although this may be a necessary first step), but to determine how to improve the standard of care in the future. If the end result is merely to identify a culpable individual, the exercise is worthless.

## 10 Standard evaluation systems

If everyone has different standards of evaluation, this obscures the bigger picture. The World Health Organization has set standards for best corrected vision at two months after surgery:

- 6/18 or better for 90% of eyes
- <6/60 for less than 5% of eyes

There are two problems associated with these guidelines. Firstly, few patients return for follow-up at two months, so the assessment of outcomes represents only a small fraction of operations. Secondly, although vision may be tested with best correction in the clinic, the patient may not buy the glasses, or the spectacles may be lost or broken within a month. Now that IOLs are almost universal and biometry is widely used, we could set standards for uncorrected vision at an earlier date — such as one week. This would allow more consistent reporting of outcomes, which would make it easier to identify best practice.

## 11 Refraction and spectacles

Even with biometry, some patients will have significant postoperative refractive error. One of the best ways to improve outcomes is to perform refraction for all patients and to give them spectacles. If there is significant astigmatism, the spectacles may be more expensive than the surgery, as astigmatic lenses are costly to prescribe and fit. Since most surgeons are aiming for good uncorrected vision, we should give spectacles either free of charge or for a minimal fee, to any patient who requires spectacles to achieve 6/18 or better.

## 12 Understanding our limitations

We have emphasised cataract surgery, as this is the most common procedure undertaken by ophthalmologists. However, the proposals are applicable to any simple or complex eye operation. In high-income countries, ophthalmologists often specialise, for example in vitreoretinal surgery. General ophthalmologists perform most common procedures, but they refer complex problems, such as paediatric cataract, to a sub-specialist colleague. In developing countries, it can be difficult to establish such a referral network: travel is costly and difficult for patients, and people prefer to deal with the doctor they know and trust rather than visit an unknown surgeon in a distant place. However, the outcomes of surgery for these complex conditions always improve when patients are referred to specialists who have the necessary equipment, training, and personnel to obtain the best results.

## 13 Leadership

This is perhaps the most important point. If the quality of outcomes is seen purely as the job of the ophthalmologist, then it is unlikely that the results will ever improve. Every eye worker has to be involved, because every stage of the patient's journey, from diagnosis to discharge, can affect the outcome. This includes not only doctors and nurses, but also non-clinical staff, such as administrators and technicians. The surgeon's role is to provide leadership and to involve all the other personnel in ensuring that every patient gets the best treatment. A change in attitudes will be accomplished by involving all health workers and allied personnel in partnership, not by giving lectures or orders from above.

On the back page of this journal, you will find the CBM logo with the motto: “Together we can do more.” This is the best advice you can ever follow, if you want to improve the quality of your surgical outcomes.

**Resources for improving outcomes**

1. The **free software package** ‘Monitoring Cataract Surgical Outcomes’ (MCSO) can be downloaded from: www.iceh.org.uk/display/LIB/Software+-+Monitoring+Cataract+Surgical+Outcomes
  For a physical copy, you can order the ‘Community Eye Health Updates 2007’ CD from TALC, PO Box 49, St Albans, Hertfordshire, AL1 5TX, UK. Email: info@talcuk.org Website: talcuk.org
2. **Instrument maintenance training** is available at low cost at Aravind Eye Hospitals in India — see www.aravind.org

## References

[1] Limburg H, Foster A, Vaidyanathan K, Murthy GV (1999). Monitoring visual outcome of cataract surgery in India. Bull World Health Organ.

[2] Yorston D (2008). Using intracameral cefuroxime as a prophylaxis for endophthalmitis. Community Eye Health J.

[3] Limburg H, Foster A, Gilbert C, Johnson GJ, Kyndt M, Myatt M (2005). Routine monitoring of visual outcome of cataract surgery. Part 2: Results from eight study centres. Br J Ophthalmol.

[4] Yorston D, Gichuhi S, Wood M, Foster A (2002). Does prospective monitoring improve cataract surgery outcomes in Africa?. Br J Ophthalmol.

